# Informatic application to characterise and identify small mammal species: Arvicolinae (Cricetidae, Rodentia, Mammalia)

**DOI:** 10.1002/ece3.70064

**Published:** 2024-09-18

**Authors:** M. P. Alfaro‐Ibáñez, E. Angel‐Beamonte, A. C. Domínguez‐García, G. Cuenca‐Bescós

**Affiliations:** ^1^ Aragosaurus‐IUCA, Departamento Ciencias de la Tierra, Facultad de Ciencias Universidad de Zaragoza Zaragoza Spain; ^2^ Aragosaurus Zaragoza Spain; ^3^ Departamento de Geodinámica, Estratigrafía y Paleontología, Facultad de Ciencias Geológicas Universidad Complutense de Madrid Madrid Spain

**Keywords:** Arvicolinae, informatic application, MatLab, quaternary, systematics

## Abstract

The classification of rodent species can be challenging due to high morphological similarities observed among them. This problem is further increased in palaeontological systematics, where classification is traditionally based on the molar morphology. The subfamily Arvicolinae (Rodentia, Mammalia) is one of these rodent groups, whose classification being important for biostratigraphic and climatic studies of the Quaternary period is challenging. We present an application developed using the MatLab informatic algorithm, designed to classify the Arvicolinae species using Geometric Morphometrics (GMM) analyses of the first lower molar. Moreover, the application includes an option to automatically obtain the linear measurements that are commonly used for the identification of these species. This method shows a high degree of accuracy in the species classification, which is expected to increase as the reference database is further developed. This application can serve as an alternative tool for the classification of specimens with unclear morphologies. It can also be used to reduce the time required to manually obtain the linear indices necessary for their classification.

## Graphical abstract

We have developed a new application in the MatLab environment to address the challenging classification of Arvicolinae species, using images of the first lower molar, achieving high accuracy in the species classification.

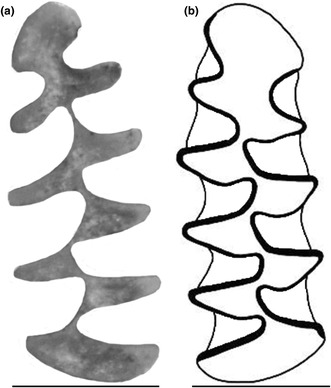

## 1 INTRODUCTION

The subfamily Arvicolinae includes voles and lemmings (Cricetidae, Rodentia, Mammalia), with a total of 161 extant species according to Pardiñas et al. (2017). The taxonomic classification of these species is challenging due to convergent evolution and significant morphological similarities characteristics of these rodents, despite their presence across a wide range of Holarctic habitats (e.g., Chaline et al., 1999; Kryštufek & Shenbrot, 2022). This complexity is also observed in the palaeontological systematics, which relies on the morphology and size of the occlusal surface of the molars (e.g., Chaline, 1972; Fejfar et al., 2011; van der Meulen, 1973), as these are generally the only anatomical part, or the best preserved, of small mammal remains after the taphonomic process.

The high number of species and genera, their adaptations to different habitats and their high diversification and evolutionary rates have made this subfamily of small mammals one of the most useful tools for Quaternary studies. Accurate classification to the species level is therefore necessary to study the climatic and biostratigraphic variations in the Quaternary record (e.g., Alfaro‐Ibáñez et al., 2023; Baca et al., 2022; Cuenca‐Bescós et al., 2016; Lemanik et al., 2022; López‐García et al., 2021).

Due to the complexity and morphological similarities of the molars, different methodologies have been developed for the study of this structure. These include the study of distinctive morphological features (e.g., Chaline, 1972; Cuenca‐Bescós & Morcillo‐Amo, 2022; Luzi, 2018) and numerous works demonstrating the applicability of Geometric Morphometrics (GMM) analysis to this subfamily of rodents (e.g. Alfaro‐Ibáñez et al., 2023; Arbez et al., 2024; Escudé et al., 2013; Killick, 2012; Piras et al., 2012). Additionally, traditional morphometric techniques, such as the linear indices defined by Van der Meulen (1973), are commonly used.

**FIGURE 1:**
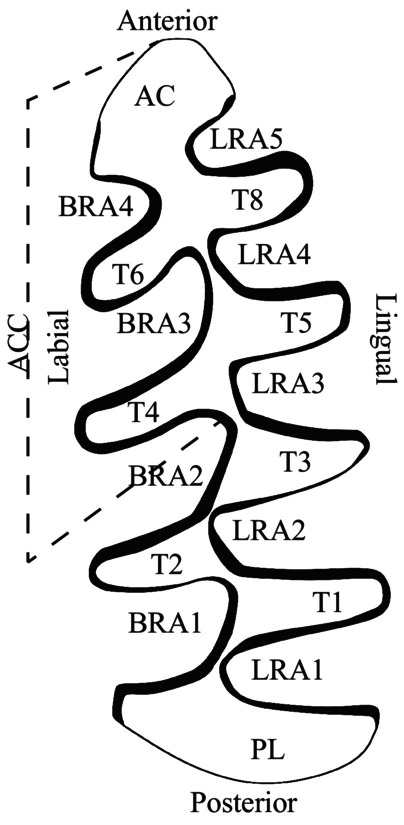
Nomenclature and orientation of the m1 of Arvicolinae species. AC, Anterior cap; ACC, Anteroconid complex; BRA, Buccal re‐entrant angles; LRA, Lingual re‐entrant angles; PL, Posterior lobule; T, Triangle.

In this work, we present an innovative methodology to overcome the taxonomic difficulties among extant and Pleistocene species of Arvicolinae in Europe. This methodology is based on the differentiation and classification of the Arvicolinae species using GMM techniques for the analysis of the occlusal surface of the molar, combined with various mathematical functions and analyses for their classification. Moreover, it includes an automatic process to measure the linear indices. The systematics of fossil arvicolines is predominantly based in the occlusal surface of the molars and the scientific literature is replete with drawings (such as the one in Figure 1) and/or photographs of this anatomical part of the rodent molar. Until now, there has been no automatic or informatic methodology that allows for the analysis of drawings and photographs from scientific papers in an automatic way. Similar studies have been conducted with other taxa, highlighting the usefulness of such methodologies (e.g., machine learning combined with GMM) in the classification of different species with problematic taxonomic differentiation (e.g., Ángel‐Beamonte et al., 2018; Domínguez‐García et al., 2024; Miele et al., 2020; Moclán et al., 2023; Quenu et al., 2020).

We have developed an informatic procedure, registered at UNIZAR (PII‐2023‐0007) and available on GitHub, with the explanation of how to use it: https://anonymous.4open.science/r/m1classifierandprocessing‐13FD. The program, including the intellectual property rights and all necessary information for its utilisation will be referred as the SOFTWARE.

## 2 THE SOFTWARE

**FIGURE 2:**
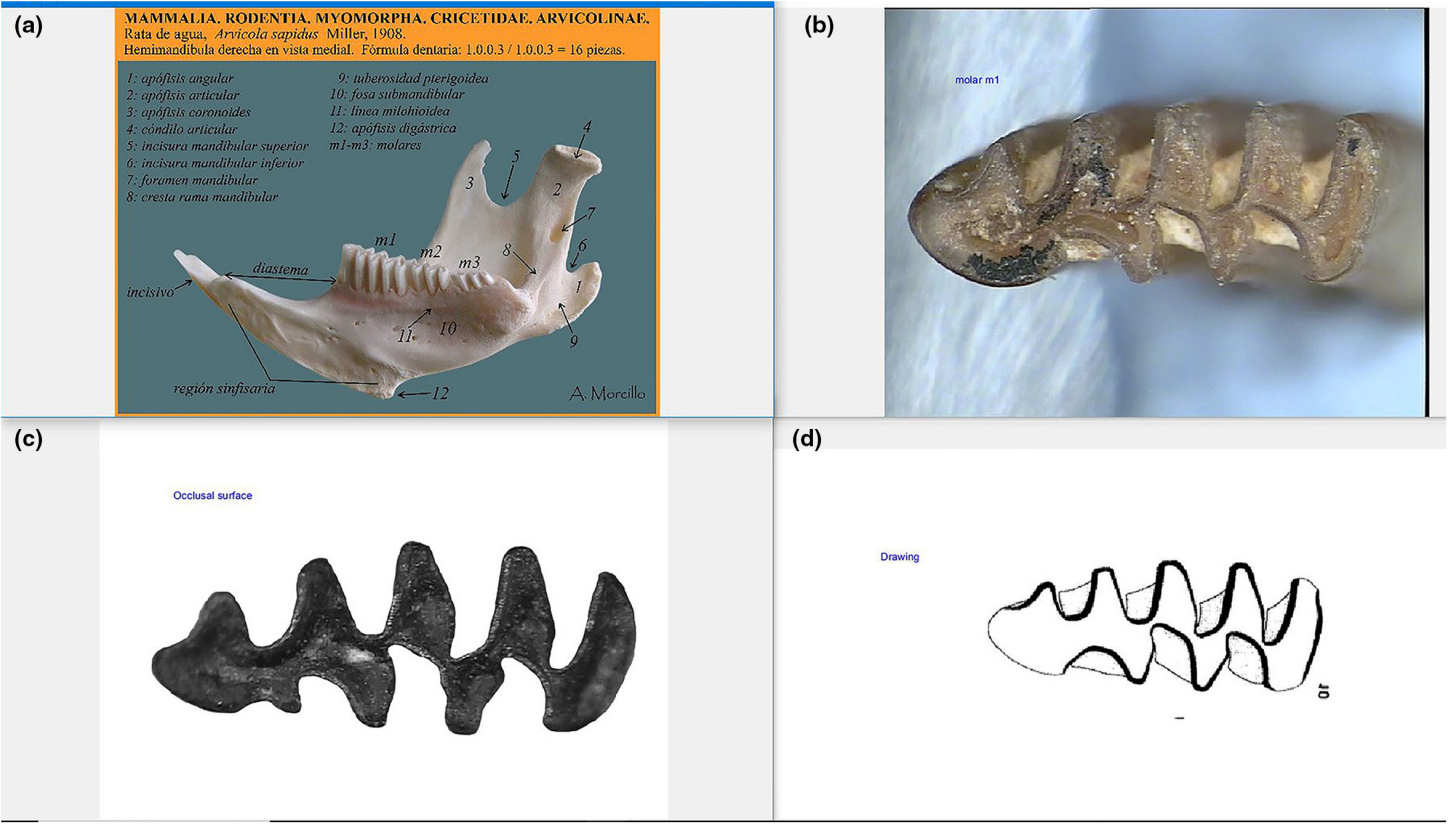
Image of the four screens that conform the environment of the SOFTWARE.

The SOFTWARE is comprises by the development of two different informatic programs that are interrelated: ‘m1processing’ and ‘m1classifier’. These applications also include an option to measure the Van der Meulen (1973) indices. The SOFTWARE is developed using graphical interfaces based on basic algorithms from Matlab R17b (The MathWorks Inc., 2017), functions of its toolboxes, additional functions developed by other developers and our own functions created specifically for these two programs. Therefore, the SOFTWARE is compatible with this version and subsequent versions of Matlab. Each of the informatic programs, once started, consists of four different screens (Figure 2):

Screen 1 (top left): shows an example of a mandible of Arvicolinae (Figure 2a).

Screen 2 (top right): shows an example of the m1 (Figure 2b).

Screen 3 (bottom left): shows an example of an image of the outline surface (Figure 2c).

Screen 4 (bottom right): shows an example of a drawing (Figure 2d).

### 2.1 Construction of the database

For the construction of the database, we collected specimens of extant and Pleistocene species of Arvicolinae from Europe. These specimens were obtained from scientific papers, modern collections and owl pellets. Most of the images of the database correspond to specimens from the Iberian Peninsula. As demonstrated by Killick (2012), there is a morphological difference between modern and fossil representatives of Arvicolinae species. Therefore, we included both fossils and modern specimens in our database with. A total of 464 first lower molars (m1) belonging to the following species were included: *Microtus arvalis*, *Microtus agrestis*, *Chionomys nivalis*, *Iberomys cabrerae*, *Stenocranius gregalis*, *Alexandromys oeconomus*, *Terricola lusitanicus*, *Terricola duodecimcostatus* and *Terricola pyrenaicus*. We also added to the database 50 specimens of the Early Pleistocene fossil genus *Allophaiomys* from the site of La Sima del Elefante (Sierra de Atapuerca, Burgos, Spain). The origin of each image is show in the Appendix S1.

#### 2.1.1 m1processing: Collection and processing of the images

The SOFTWARE works with images of the occlusal surface of the m1. These images were obtained using a stereographic microscope equipped with a digital camera, thus obtaining photographs of both fossil and modern specimens. In addition, some images were obtained from published scientific papers. The selection criteria for these images and drawings has been to include specimens in which the complete outline of the occlusal surface is clearly visible. Specimens from published scientific papers were also required to have enough quality, scale and identification to the species level to be selected for the database.

**FIGURE 3:**
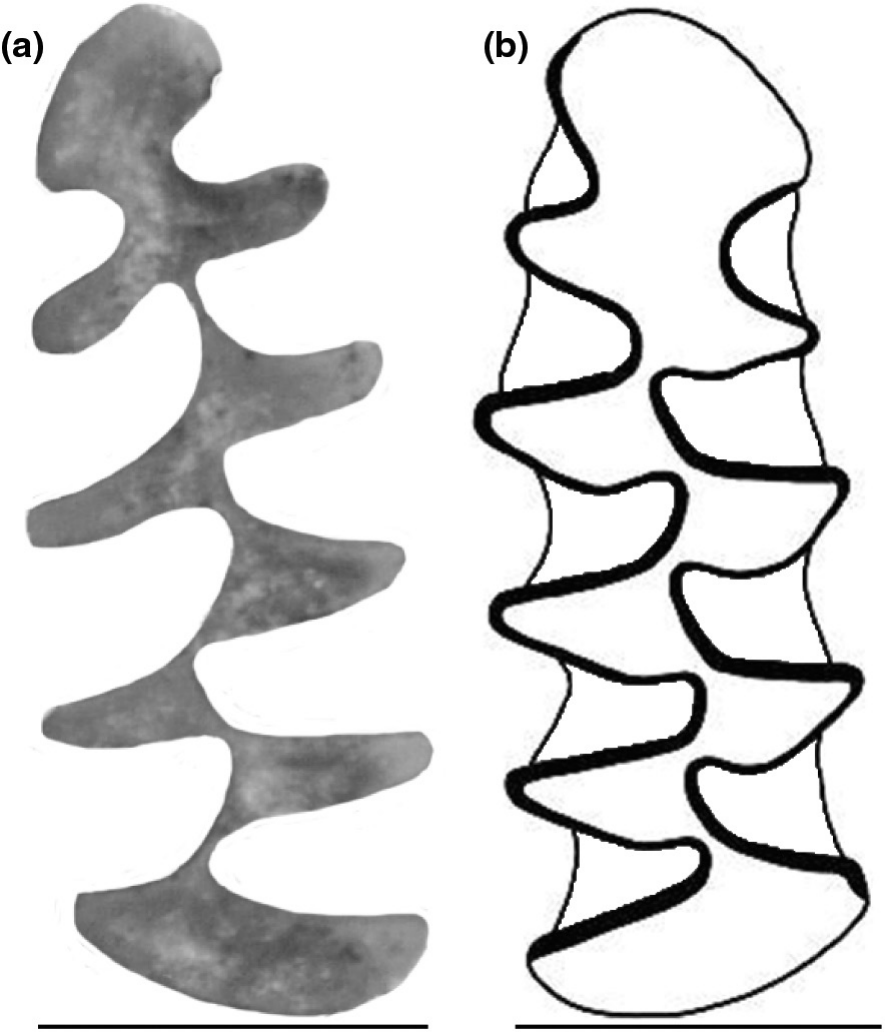
Examples of the images (a) and drawings (b) types of images, prepared to be characterised by the different applications of the SOFTWARE. (a) *Terricola pyrenaicus*, edited photography from El Miron Cave, level 128, spit 14, subsquare C, square X10, (b) *Microtus arvalis* drawing modified from Luzi (2018).

The backgrounds of the images and drawings obtained from other bibliographical databases need to be removed before their incorporation into the SOFTWARE (Figure 3), for the program to be capable of accurately identify the outline. These images should correspond to the outline of the molar on the interior of the enamel, to avoid the effects of attritional wear, possible enamel fractures, or corrosion.

**FIGURE 4:**
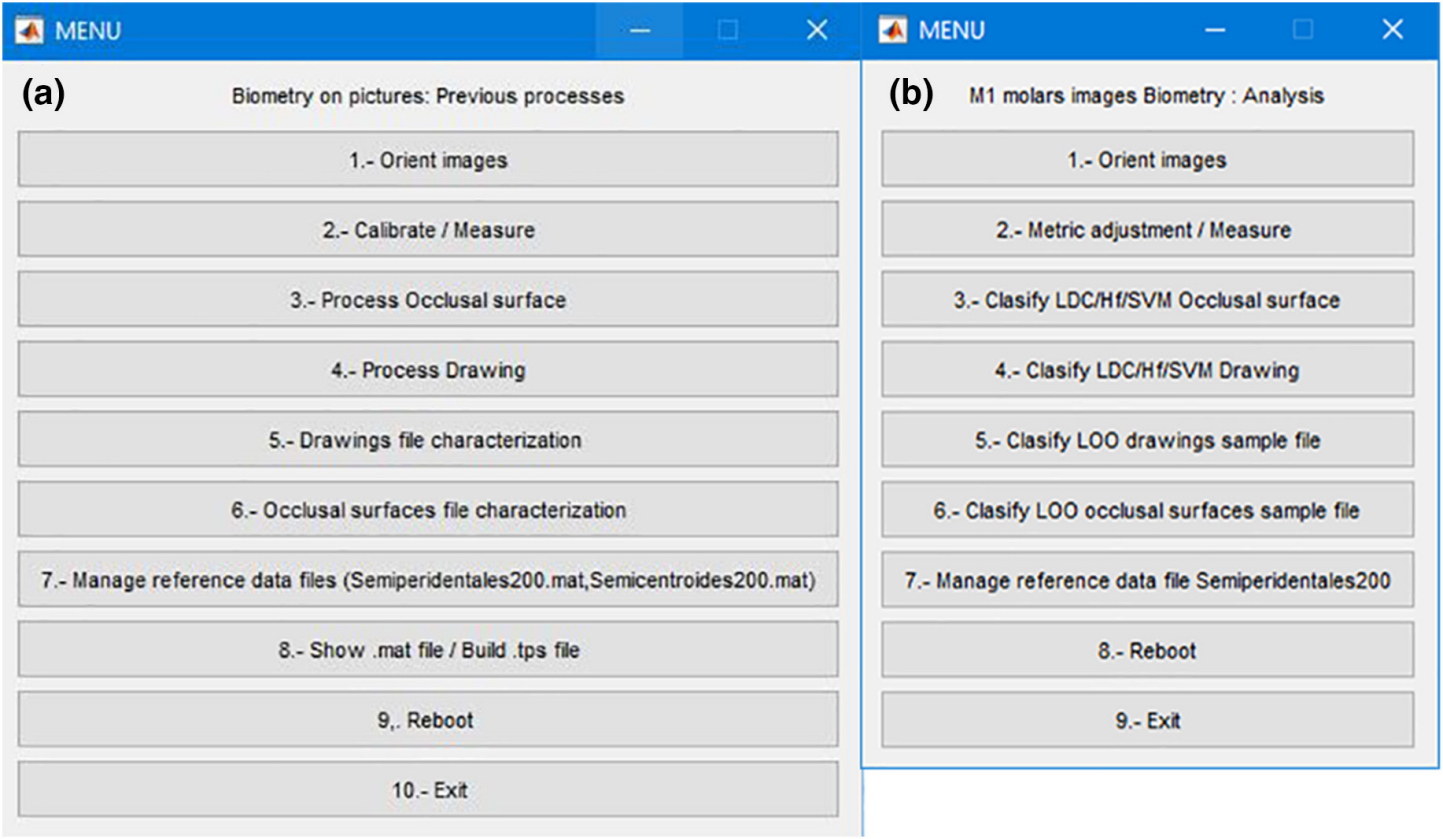
Menu screens of m1processing (a) and m1classifier (b).

**FIGURE 5:**
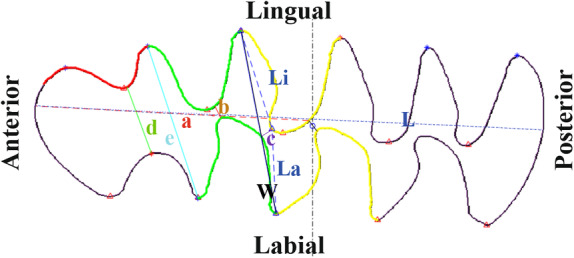
Orientation of the m1 on the SOFTWARE and the Van der Meulen (1973) indices traditionally used for the study and classification of Arvicolinae species.

The processing of the images is conducted using ‘m1_processing’, which allows normalising the resolution of the images and change the orientation of the specimens to ensure consistency in the results. All the process can be done navigating within the menu options (Figure 4). Image reorientation involves positioning the anterior part of the molar on the left of the screen and the labial part at the bottom (Figure 5). With the use of the scale of the molars, the program performs the calibration either individually or in batch mode for multiple images from the same source. Processed images were archived in new folders, according to their origin, to be part of the database of characterised individual.

To obtain the GMM data from the molar outlines, we implemented an algorithm in the program, applicable both individually and in batch mode. We obtained the outline of the occlusal surface of the first lower molar (m1) (Figure 1) from the interior part of the enamel to avoid the effects of attritional wear, allowing us to analyse even bibliographic materials.

Images and drawings are processed using slightly different methods due to the characteristic of each type of format. For images, the program directly identifies the outline of the molar. However, for drawings, the program is able to remove the represented cement areas and identifies the molar outline as the middle part of the drawing line (Figure 3). The outline is represented by a white line, of one pixel wide with a logic value of ‘1’ for each pixel, on a black background with a logic value of ‘0’ for each pixel. Another algebraic algorithm locates the anterior part of the outline, from the apex of the molar to the point that defines the segment ‘a’ according to Van der Meulen (1973) between T4 and T3 (Figure 5). This anterior part comprises the triangles T4 to T7 and the AC. We have excluded the posterior part of the molar for this process as it does not present relevant information for this type of characterisation.

Once the semiperimeter is obtained, another algorithm records the number of white pixels and performs a uniform decimation along the pixels of the semiperimeter curve. In this way, the number of pixels of the semiperimeter that represent the individual is reduced to 200. This number of landmarks was determined empirically: using 100 points did not provide accurate discrimination among the different species, while increasing to 300 points the classifications obtained did not improve. In addition, a Fourier analysis indicated that a density of 100 landmarks would meet the Nyquist criterion for the correct representation of the molar profile in subsequent reconstructions. The database is made up of the information from each sample, including corresponding species and the coordinates of the 200 landmarks of the semiperimeter. This information of each specimen is stored in a file named ‘Semiperidentales200.mat’. Using this file, the program is able to generate another one containing the information of the coordinates of the 200 points of the centroids of each species, named ‘Semicentroides200.mat’.

### 2.2 m1classifier: Affinity estimation

In the same way that with m1porcessing, all the tasks can be done by navigating within the menu options (Figure 4). To estimate the affinities between the samples under study and the reference database, we use the second program, ‘m1classifier’. We implemented four different methods for estimating affinities: support‐vector machine (SVM) (Boser et al., 1992; Carmona Suárez, 2014; Cortes & Vapnik, 1995; Guyon et al., 1993; Smola, 1996), Hausdorff distance (HD) (Dubuisson & Jain, 1994; Sasikanth, 2022), Procrustes distance (Gower, 1975) and Fisher Linear Discriminant analysis (Fisher, 1936; Peña, 2002). These classification processes can be carried out for one specimen or in batch mode for the automatic estimation of various images, previously oriented and calibrated with the program ‘m1_processing’.

#### 2.2.1 Support vector machine

The SVM, also known as support‐vector network or support‐vector machine, is a methodology that works under the premise of reducing structural risk. among other uses, it is widely used for image recognition challenges and performs effectively on smaller, cleaner datasets compared to other methodologies.

The basic idea of SVMs, just like 1‐layer or multi‐layer neural networks, relies on finding an optimal hyperplane for linearly separable patterns and extend this to patterns that are not linearly separable by transformations of the original data to map it into new space by means of a Kernel function. The decision function is fully specified by a (usually very small) subset of training samples known as support vectors.

SVM analysis generates hyperplanes equidistant from the two most similar specimens of two different species in the database. These specimens are considered support vectors (Carmona Suárez, 2014; Cortes & Vapnik, 1995). Support vectors are the data points nearest to the decision surface (or hyperplane) and have the following properties (Nefedov, 2016):

- They are the data points most difficult to classify.
- They have direct bearing on the optimum location of the decision surface.

SVM finds an optimal solution by maximising the margin around the separating hyperplane, a task that involves solving a quadratic programming problem. This problem is easy to solve by optimisation techniques, specifically through the application of Lagrange multipliers to get this problem into a form that can be solved analytically. In other words, the Optimal Hyperplane is the specific hyperplane for which the margin of separation between classes is maximised.

Under Matlab there is a group of functions dealing with SVMs. Since SVMs works only on binary classification problems (i.e., only two response classes), to perform a multiclass classification we must use a combination of binary SVMs. We have implemented our SVM algorithm using the Matlab toolbox function ‘fitcecoc’, which is intended to find multiclass models for support vector machines or other classifiers.

Mdl = fitcecoc(*X*,*Y*) returns a trained ECOC model (compact multiclass error. correcting output codes) Mdl using the predictors *X* and the class labels *Y*. This approach allows for multiclass learning by reducing the model to multiple binary classifiers, such as support vector machines. Then we confront the Mdl model with new data to achieve an optimal class estimation using the ‘predict’ function.

ECOC models integrate misclassification costs by incorporating them with class prior probabilities (which are uniform in our case). The SOTWARE adjusts the class prior probabilities and sets the cost matrix to the default cost matrix for binary learners.

#### 2.2.2 Hausdorff distance

The HD is a mathematical construction to measure the ‘proximity’ of two sets of points that are a subset of one metric space. This measure provides a scalar grade to the similarity between two sets of points. An improvement in this function is the modified Hausdorff distance (MHD) (Sasikanth, 2022), which has shown to work better than the HD in distance calculations (Dubuisson & Jain, 1994). The implementation in our program have been done using the algorithm ‘vectorised _MHD’, developed by González Olmos (2018) for Matlab. This algorithm allows us to directly use the two‐dimensional spatial coordinates of the 200 points of the semi‐perimeter of the m1. Our program enables the execution of a Hausdorff analysis for each of the characterised specimen (HDV).

#### 2.2.3 Procrustes distance

**FIGURE 6:**
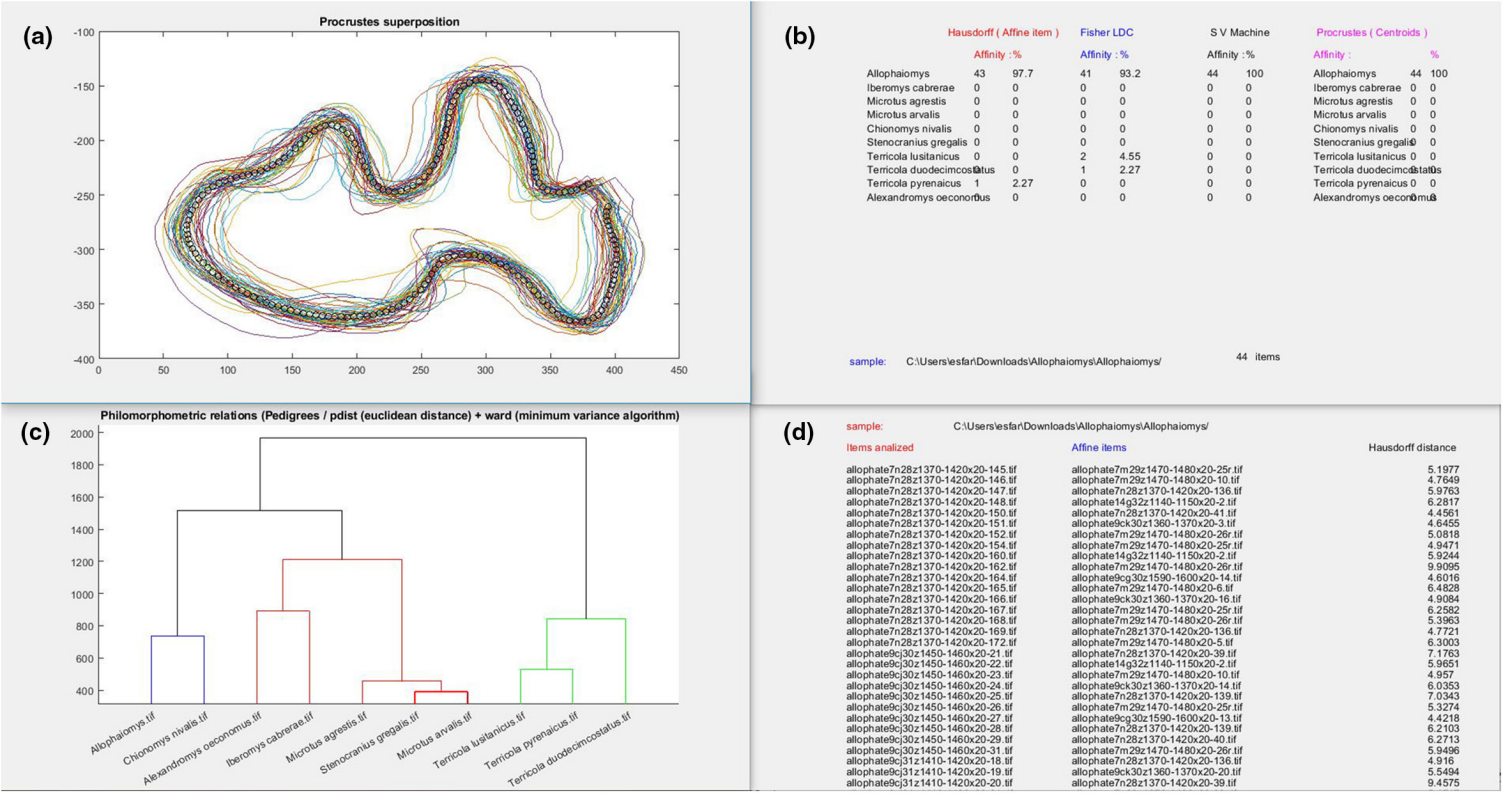
Example of a LOO analysis of the images of the occlusal surfaces of *Allophaiomys* sp. not included in the reference database of the program. (a) m1 outlines of the analysed *Allophaiomys* sp. according to Procrustes analysis (b) Classification obtained by HD, Procrustes, Fisher LDC and SVM, (c) Cluster of the different the species of the database, (d) More similar individual of the database of each one of the specimens analysed by Procrustes.

The Procrustes analysis (Gower, 1975) quantifies the geometric affinity between two data sets. In the data used by the SOFTWARE, these data sets are constituted by the Cartesian coordinates of the 200 points that define the semi‐perimeter. Given two matrices of identical size, A, B, Procrustes analysis standardised both in a way that (𝐴𝐴)^𝑇^=1 and centres both sets of points around the origin. Subsequently, the optimal transformation is applied to the second matrix (scale, rotation and reflexion) to minimise the sum of the square of the pointwise differences between the two set of data. The result of these transformations is displayed when a batch analysis is conducted, allowing us to visualise and compare the outlines of each m1 present in the analysed file (Figure 6a). This algorithm is implemented in the function ‘procrustes’ of the Matlab toolbox.

#### 2.2.4 Fisher linear discriminant analysis

The linear discriminant function deduced by Fisher (LDC) for various different groups (N) is based on the finding of the canonical variables with the highest discriminant power to classify new specimens among the originally defined N varieties (Peña, 2002). The objective of this function is to define a vector of *q* canonical variables, with *q = min*(*N‐1,p*), being *p* the number of original variables (the vectors with the 200 coordinates of the pixels of the anterior semi‐perimeter of the previously identified specimens), that we obtain as a linear combination of the original variables. These canonical variables *q* let to address the problem of the classification of the samples by three successive phases:

1. The measurements of the original variables are projected for the different varieties in the space determined by the *q* canonical variables
2. The vector of the semi‐perimeter coordinates of the individual or specimens to be classified is projected into the same canonical variable space
3. The function classifies the specimens in which species between the *N* would be more similar, using the Mahalanobis distance in the space of the canonical variables

The Fisher discriminant analysis is executed using the algorithms ‘*fitcdiscr*’ and ‘*predict*’ in Matlab. The first one deduces the canonical variables with the highest discriminant power and obtains the projections of the coordinates representative of the species. The second algorithm then projects the coordinate vector of the specimen onto these canonical variables and deduce the species more similar with the criterium of the Mahalanobis distance.

### 2.3 Automatic measurement of the Van der Meulen indices

**FIGURE 7:**
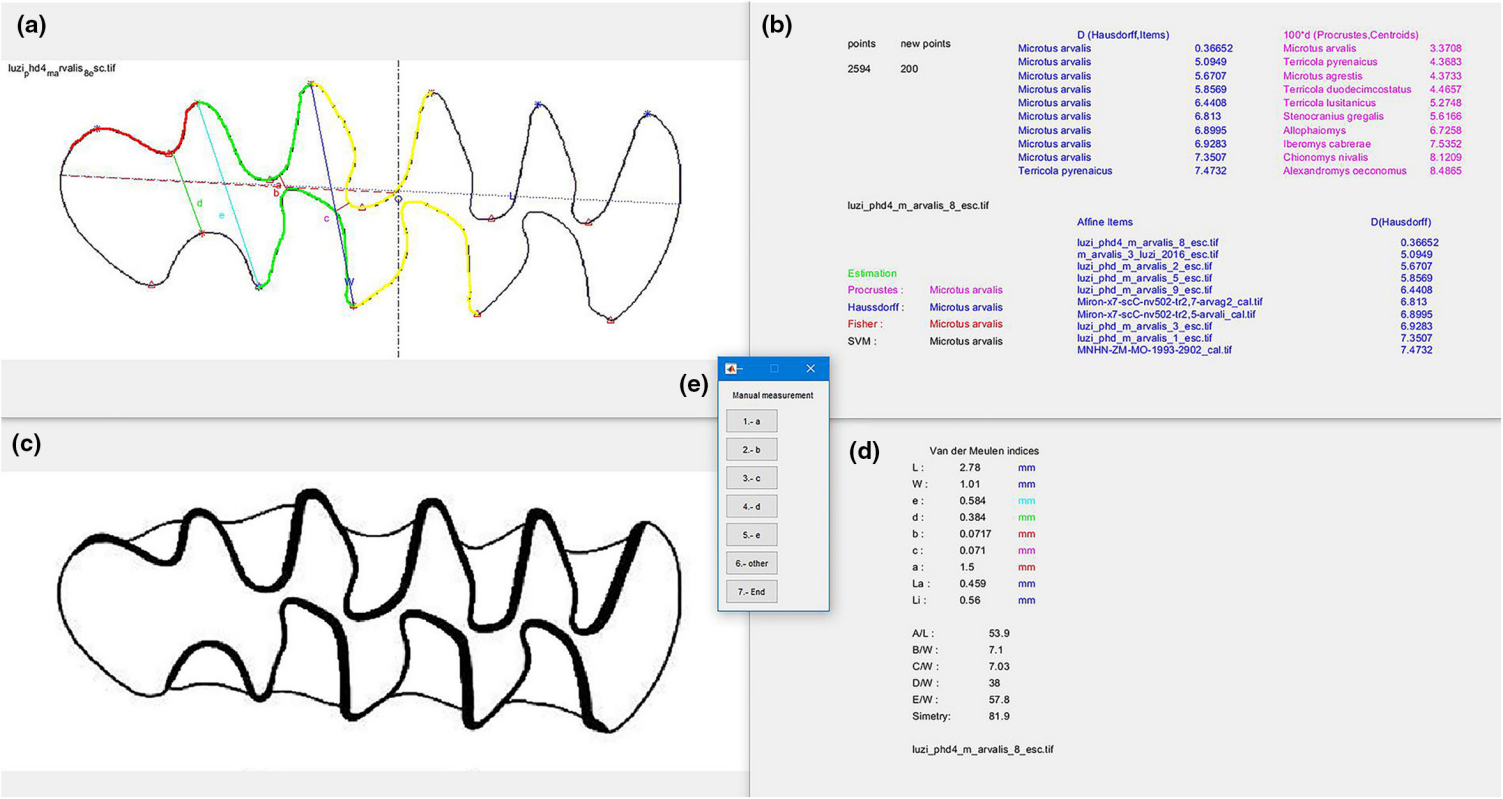
Example of one individual of *M. arvalis* (c) analysed by the SOFTWARE that shows the classification obtained (b), the perimeter and point of measure of the van der Meulen indices (a) and the obtained measures (d). The menu (e) let us to choose what indices we want to measure manually.

The SOFTWARE enables the automatic calculation and automatically represent the Van der Meulen indices (Van der Meulen, 1973) for each specimen, as well as the asymmetry measurements of the molar (La and Li) as indicated by Cuenca‐Bescós et al. (1995). The SOFTWARE automatically displays these indices in the screen 1 (Figure 7a), with their numeric values shown in the screen 4 (Figure 7d). For the calculation of the Van der Meulen indices, the SOFTWARE retrieves the information of the posterior part of the m1. This same option allows users to manually measure the indices directly from the original image, in the screen 3 (Figure 7c). Once manually measured, the SOFTWARE automatically recalculates the rest according to the redefine parameter.

## 3 ACCURACY OF THE ANALYSIS

### 3.1 Affinity estimation

For studying the effectivity of the different analytical methods, we conducted a batch analysis of each species in the database using the method Leave One Out (LOO). This method allows us to compare one specimen from the database with the remaining database. Additionally, we analysed 44 images of specimens identified as *Allophaiomys* sp. from La Sima del Elefante (Sierra de Atapuerca, Burgos, Spain), not included in the database, to assess the discriminatory power of the program.

The program returns the classification of each specimen, in the screen 2 (Figure 6b), indicating at which species each m1 is most similar, according to each classification method (Fisher LDC, SVM and Procrustes). It also identifies the specimen of the database that has the closest morphology to the analysed one according to the Procrustes, shown in the screen 4 (Figure 6d).

The program also constructs a cluster using one model specimen from each species that is correctly classified by all methods of analysis (Figure 6d). However, this cluster does not have any biological or phylogenetic significance. It only provides information on the geometric similarities of the species within the SOFTWARE, thus indicating potential misclassifications.

In general, we observed that among the three species with less than 50 characterised specimens, the lowest accuracy values are obtained to classify *Terricola lusitanicus* and *Terricola pyrenaicus* (73,8% and 60,7% respectively). The Hausdorff analysis and SVM provided better accuracy, while LDC yielded the lowest accuracy results (Table 1).

**TABLE 1:** Accuracy for each species of the HD V, Procrustes, Fisher linear discriminant (LDC) and SVM analyses.

Sample	Images	HD V	Procrustes	LDC	SVM	Drawings	HD V	Procrustes	LDC	SVM	TOTAL	HD V	Procrustes	LDC	SVM
*Allophaiomys* sp.	50	48	48	43	48	0	0	0	0	0	50	96.0%	96.0%	86.0%	96.0%
*Iberomys cabrerae*	35	34	21	15	34	17	15	15	8	17	52	94.2%	69.2%	44.2%	98.1%
*Microtus agrestis*	29	25	21	16	27	21	13	8	4	16	50	76.0%	58.0%	40.0%	86.0%
*Microtus arvalis*	23	22	21	9	21	28	21	16	16	26	51	84.3%	72.5%	49.0%	92.2%
*Chionomys nivalis*	21	20	18	16	20	29	26	11	17	27	50	92.0%	58.0%	66.0%	94.0%
*Stenocranius gragalis*	27	24	20	17	26	23	19	18	14	22	50	86.0%	76.0%	62.0%	96.0%
*Terricola lusitanicus*	36	25	28	11	27	6	4	3	1	3	42	69.1%	73.8%	27.9%	71.4%
*Terricola duodecimcostatus*	28	22	17	13	19	22	15	18	7	18	50	74.0%	70.0%	40.0%	74.0%
*Terricola pyrenaicus*	21	15	16	5	13	6	1	1	2	4	28	57.2%	60.7%	25.0%	60.7%
*Alexandromys oeconomus*	22	22	22	18	22	20	17	16	11	18	42	92.9%	90.5%	69.2%	95.2%

*Note*: In dark grey the highest accuracy for each species, in light grey the second highest.

The classification of the 44 additional images of *Allophaiomys* sp. showed maximum accuracy of 100% with SVM, with only one specimen misclassified using the HD (Figure 6).

### 3.2 Linear measurements: Automatic vs manual calculation

We compared the measurements obtained by the program with those obtained manually on a total of 30 specimens (18 drawings and 12 images) (Appendix S2). The Van der Meulen indices and symmetry indices were measured a total of 20 times on one individual to corroborate that the differences between the manual and automatic measurements are not statistically significant (Appendix S3). Manual measurements were made on the digital images with an image editing program.

The automatic measurements of the Van der Meulen (1973) indices and the asymmetry showed a total mean discrepancy ranging between 0.006 mm and 0.022 mm for the different indices compared to the manual measurements, with no significant differences between the discrepancies for drawings and images (Appendix S2). We observed greater differences between the automatic and manual measurements for the parameter ‘e’, reaching a maximum difference of 0.079 mm. The highest measurement discrepancies were observed in the species *Alexandromys oeconomus* and *Chionomys nivalis*, which could be due to the morphology of the m1 of these species.

In some cases, not all the indices could be measured, as is the case for certain morphotypes of *C. nivalis*, *A. oeconomus*, or *Iberomys cabrerae*. However, the automatic measurements carried out by the program still gives a measure for the indices in these cases (Appendix S2), that should not be considered valid. Nonetheless, the remaining indices are correctly measured.

## 4 DISCUSSION

### 4.1 Accuracy rates

SVM has proven to be the most accurate discriminant analysis method within this program, followed by HDV and could lead to dispense the use of the Fisher LDC, Procrustes and HDC.

Fisher LDC has proven to be the less efficient of the analyses, being the only one that applies a linear classification approach using canonical variables to classify the species (Table 1). Furthermore, SVM offers several key advantages over Fisher LDC analysis: 1. Flexibility, SVM does not assume a specific data distribution, making it more flexible and robust; 2. Handling non‐linear boundaries, while Fisher LDC is constrained to linear boundaries; 3. Sparsity and efficiency of the SVM compared to LDC, due to SVM's solutions depend on the support vectors; and 4. Handling high‐dimensional data, due to SVM only depends on the number of support vectors rather than the dimensionality, whereas LDC can struggle with the curse of dimensionality, as the number of parameters to estimate increases rapidly (e.g., Lee, 2010; Obi, 2017). Thus, SVM provides a more flexible, efficient and robust classification approach compared to the classical LDC method, especially for complex, high‐dimensional or non‐linear data. SVM is more suitable than LDC for non‐linear classification problems and handling high‐dimensional data with non‐normal distributions.

Comparing the accuracy between the two most precise methods, HDV and SVM, we observed that in general, SVM shows a higher discriminant power (Table 1). SVM shows an accuracy above 60% across all species in the database, achieving the highest accuracy in almost all species, except for *Terricola lusitanicus*, which shows the highest accuracy with Procrustes analysis. Between the HDV and Procrustes analysis, HDV generally shows better accuracy than Procrustes, but the latter shows higher accuracy in the case of three species (*Allophaiomys* sp., *T. lusitanicus* and *T. pyrenaicus*), whereas HDV only for two of them (*Allophaiomys* sp. and *T. duodecimcostatus*). The species for which the SOFTWARE display the lowest discriminatory power correspond to those with the smallest number of specimens in the database, particularly *T. pyrenaicus*, with only 27 specimens and an accuracy of 60.7% using SVM and Procrustes. Observing the results for other species, it is inferred that the accuracy will increase along with the number of specimens. Notably, the lower accuracy values are observed within the *Terricola* genus. These species exhibit significant morphological similarities and high intraspecific variability in molar morphology (Román, 2019), which may contribute to the slightly lower discriminatory power between these species. Another pair of species which also display similar morphology and size are *Microtus arvalis* and *M. agrestis* (e.g., Abbassi, 1998; Luzi, 2018; Luzi & López‐García, 2017), which the SOFTWARE was able to differentiate with an accuracy of 92.2% for *M. arvalis* and 86% for *M. agrestis*. These percentages are similar to the ones obtained by Navarro et al. (2018), considering that our database of reference also included fossil samples, which exhibit greater morphological similarity than modern representatives, as also observed by Navarro et al. (2018) and Killick (2012).

Analysing a subset of 44 images of *Allophaiomys* sp., all of them different of the references included in the database of this species, the SOFTWARE demonstrated high discriminatory power, with a 100% of accuracy using SVM analysis. In this way, the SVM demonstrated again to be the most efficient classification analysis in the SOFTWARE.

### 4.2 Differences between automatic and manual linear measurements

The developed option to automatically calculate the asymmetry and the parameters of Van der Meulen has shown minimal discrepancies when compared with manual measurements of the same parameters. In any case do the differences exceed 1 mm and, in general, they do not exceed 0.5 mm. Examining the mean differences of each parameter, none reaches the 0.4 mm. At the same time, no significant differences were observed between the mean discrepancies of measurements between images and drawings (S.2), indicating consistent algorithm performance in both cases. The cases where discrepancies between the two measurement methods are more significant could be attributed to the specific morphology of the m1 in those specimens or species. For the parameter ‘e’, the program measures it as the distance between the points of major curvature of T6 and T7. It is encoded in the algorithm as the distance between the lowest point of T6 and the highest point of T7, considering the orientation of the m1 in the program. For this reason, species like *C. nivalis* and *A. oeconomus*, which exhibit morphologies where T6 is absent, as in *A. oeconomus*, or with a pronounced inclination together with T7 towards the posterior part of the molar (e.g. Chaline, 1972; Nadachowski, 1991), could generate discrepancies compared to manual measurements. However, the SOFTWARE facilitates manual correction of automatic segment measurements found to be erroneous.

## 5 CONCLUSIONS

The SOFTWARE, along with all the algorithms it includes, is specifically designed for the study of Arvicolinae species. Therefore, the application of the program for the analysis of additional species from other subfamily of rodents or mammals with different molar structures, would require modifications of the codes and algorithms of the SOFTWARE.

Despite the need to expand the sample size of some species in the database, the SOFTWARE, demonstrates a generally high discrimination power between species with similar morphological characteristics of the m1. The SVM has shown the highest discriminating power, allowing to differentiate between overlapping morphologies (as *M. arvalis* and *M. agrestis*). The HD also exhibits substantial discrimination capacity, while Fisher LDC shows the lowest accuracy. Therefore, this program is particularly useful in cases where classification is challenging due to morphological similarities between species. It can automatically deduce the taxonomic classification of the sample or obtain the measurements traditionally used for their identification, avoiding alterations of the molar as attritional wear or breaks on the enamel. In this way, the use of this program could lead to a decrease in the time needed to classify these controversial specimens, or to acquire all necessary linear measurements for taxonomic purposes and biometric characterisation.

## AUTHOR CONTRIBUTIONS

**M. P. Alfaro‐Ibáñez:** Conceptualization (equal); investigation (equal); methodology (equal); resources (equal); software (supporting); writing – original draft (lead); writing – review and editing (equal). **E. Angel‐Beamonte:** Formal analysis (equal); investigation (equal); methodology (equal); software (lead); writing – original draft (equal); writing – review and editing (equal). **A. C. Domínguez‐García:** Investigation (supporting); resources (equal); writing – review and editing (equal). **G. Cuenca‐Bescós:** Conceptualization (equal); investigation (equal); methodology (equal); resources (equal); supervision (lead); writing – original draft (equal); writing – review and editing (equal).

## FUNDING INFORMATION

This work was supported by the Government of Aragón‐ERDF (Group E18: Aragosaurus: Recursos Geológicos y Paleoambientales). M.P.A.I. is supported by a grant from the Ministerio de Universidades of Spain (FPU20/02031). A.C.D.G is a beneficiary of a Margarita Salas postdoctoral contract (CT18/22) funded by the European Union ‘NextGenerationEU/PRTR’ and received support from the European SYNTHESYS+ grant (FR‐TAF_Call4_007) and from the Complutense University of Madrid and Banco Santander by a Research Stay Grant (UCM2020‐EB25/20).

## CONFLICT OF INTEREST STATEMENT

The authors declare that there are no conflicts of interest.

### OPEN RESEARCH BADGES

This article has earned Open Materials and Preregistered Research Design badges. Materials and the preregistered design and analysis plan are available at [[insert provided URL(s) on the Open Research Disclosure Form]].

## Supporting information


Appendix S1.

Appendix S2.

Appendix S3.

## Data Availability

The SOFTWARE is available on GitHub: https://anonymous.4open.science/r/m1classifierandprocessing‐13FD. The database raw images would be available under request.

## References

[ece370064-bib-0001] Abbassi, M. (1998). Essai de différenciation entre Microtus arvalis et Microtus agrestis à partir de l'étude de quatre populations fossiles (Sud‐est de la France et Ligurie italienne). Bulletin du Museum d'Anthropologie et de Préhistoire de Monaco, 39, 45–51.

[ece370064-bib-0004] Alfaro‐Ibáñez, M. P. , Cuenca‐Bescós, G. , Bover, P. , González‐Morales, M. R. , & Straus, L. G. (2023). Implications of population changes among the Arvicolinae (Rodentia, mammalia) in El Mirón cave (Cantabria, Spain) for the climate of the last C. 50,000 years. Quaternary Science Reviews, 315, 108234. 10.1016/j.quascirev.2023.108234

[ece370064-bib-0005] Ángel‐Beamonte, E. , Martín‐Ramos, P. , Santolaria, P. , Sales, E. , Abizanda, J. , & Yániz, J. (2018). Automatic determination of landmark coordinates for honey bee forewing venation using a new MATLAB‐based tool. Journal of Apicultural Research, 57(5), 605–610. 10.1080/00218839.2018.1501856

[ece370064-bib-0006] Arbez, L. , Tereza, H. , Aurélien, R. , Montuire, S. , Oldrich, F. , & Horáček, I. (2024). Re‐investigation of fossil Lemmini specimens from the early and middle Pleistocene of Western and Central Europe: Evolutionary and paleoenvironmental implications. Palaeogeography, Palaeoclimatology, Palaeoecology, 641, 112128. 10.1016/j.palaeo.2024.112128

[ece370064-bib-0007] Baca, M. , Popovic, D. B. , Lemanik, A. , Bañuls‐Cardona, S. , Conard, N. J. , Cuenca‐Bescós, G. , Desclaux, E. , Fewlass, H. , García, J. T. , Hadravova, T. , Heckel, G. , Horáček, I. , Knul, M. , Lebreton, L. , López‐García, J. M. , Luzi, E. , Marković, Z. , Lenardić, J. M. , Murelaga, X. , … Nadachowski, A. (2022). Ancient DNA reveals interstadials as a driver of common vole population dynamics during the last glacial period. Journal of Biogeography, 50(1), 183–196. 10.1111/jbi.14521

[ece370064-bib-0008] Boser, B. E. , Guyon, I. M. , & Vapnik, V. N. (1992). A training algorithm for optimal margin classifiers. In Proceedings of the fifth annual workshop on computational learning theory (pp. 144–152). ACM Press.

[ece370064-bib-0009] Carmona Suárez, E. J. (2014). Tutorial sobre máquinas de vectores soporte (sVM). Tutorial Sobre Máquinas de Vectores Soporte (SVM), 1, 1–12.

[ece370064-bib-0010] Chaline, J. (1972). Les rongeurs du Pleistocène moyen et supèrieur de France. Cahiers de Paléontologie CNRS, 1–410.

[ece370064-bib-0500] Chaline, J. , Brunet‐Lecomte, P. , Montuire, S. , Viriot, L. , & Courant, F. (1999). Anatomy of the arvicoline radiation (Rodentia): palaeogeographical, palaeoecological history and evolutionary data. Annales Zoologici Fennici, 36(4), 239–267. http://www.jstor.org/stable/23735732

[ece370064-bib-0011] Cortes, C. , & Vapnik, V. N. (1995). Support‐vector networks. Machine Learning, 20(3), 273–297.

[ece370064-bib-0012] Cuenca‐Bescós, G. , Blain, H. A. , Rofes, J. , López‐García, J. M. , Lozano‐Fernández, I. , Galán, J. , & Núñez‐Lahuerta, C. (2016). Updated Atapuerca biostratigraphy: Small mammal distribution and implications for the quaternary Spanish biochronology. Comptes Rendus Palevol, 15(6), 621–624. 10.1016/j.crpv.2015.09.006

[ece370064-bib-0013] Cuenca‐Bescós, G. , & Morcillo‐Amo, A. (2022). Roedores, edades y paisajes en el Cuaternario de la Península Ibérica (p. 416). Prames. Guías de la naturaleza.

[ece370064-bib-0014] Cuenca‐Bescós, G. , Sanagustín, J. I. C. , & Conesa, C. L. (1995). Los arvicólidos (Rodentia, Mammalia) de los niveles inferiores de Gran Dolina (Pleistoceno inferior, Atapuerca, Burgos, España). Spanish Journal of Palaeontology, 10(2), 202. 10.7203/sjp.24137

[ece370064-bib-0015] Domínguez‐García, Á. C. , Utge, J. , Larrue, C. , Moclán, A. , Kbiri Alaoui, M. , Rocca, E. , Carrato, C. , Callegarin, L. , De Chazelle, C.‐A. , Oueslati, T. , & Stoetzel, E. (2024). First asserted record of the house mouse in Morocco: Application of a multidisciplinary approach to the site of Rirha (5th − 1st c. BC). Archaeol. Anthropological Science, 16, 93. 10.1007/s12520-024-02002-8

[ece370064-bib-0016] Dubuisson, M. P. , & Jain, A. K. (1994). *A modified Hausdorff distance for object matching*. In ICPR94, pages A: 566‐568, Jerusalem, Israel. http://ieeexplore.ieee.org/xpls/abs_all.jsp?arnumber=576361

[ece370064-bib-0017] Escudé, É. , Renvoisé, É. , Lhomme, V. , & Montuire, S. (2013). Why all vole molars (Arvicolinae, Rodentia) are informative to be considered as proxy for quaternary paleoenvironmental reconstructions. Journal of Archaeological Science, 40(1), 11–23. 10.1016/j.jas.2012.03.003

[ece370064-bib-0018] Fejfar, O. , Heinrich, W.‐D. , Kordos, L. , & Maul, L. C. (2011). Microtoid cricetids and the early history of arvicolids (Mammalia, Rodentia). Palaeontologia Electronica, 14(3), 1–38. palaeo‐electronica.org/2011_3/6_fejfar/index.html

[ece370064-bib-0019] Fisher, R. A. (1936). The use of multiple measurements in taxonomic problems. Annals of Eugenics, 7(2), 179–188. 10.1111/j.1469-1809.1936.tb02137.x

[ece370064-bib-0020] González Olmos, A. (2018). Vectorized implementation of the modified Hausdorff distance. MATLAB Central File Exchange. Recovered September 9, 2022. https://www.mathworks.com/matlabcentral/fileexchange/69335‐vectorized‐implementation‐of‐the‐modified‐hausdorff‐distance

[ece370064-bib-0021] Gower, J. C. (1975). Generalized procrustes analysis.

[ece370064-bib-0022] Guyon, I. M. , Boser, B. E. , & Vapnik, V. N. (1993). Automatic capacity tuning of very large VC‐dimension classifiers. In S. J. Hanson , J. D. Cowan , & C. L. Giles (Eds.), Advances in neural information processing systems 5 (pp. 147–155). Morgan Kaufmann Publishers.

[ece370064-bib-0023] Killick, L. E. (2012). *Geometric morphometric analysis of the Microtus M1 and its application to early middle Pleistocene in the UK* (Ph.D. dissertation, Durham University). http://etheses.dur.ac.uk/3550/

[ece370064-bib-0024] Kryštufek, B. , & Shenbrot, G. (2022). Voles and lemmings (Arvicolinae) of the palaearctic region (p. 449). University of Maribor, University Press.

[ece370064-bib-0025] Lee, Y. (2010). Support vector machines for classification: A statistical portrait. In Statistical Methods in Molecular Biology (pp. 347–368). Humana Press. 10.1007/978-1-60761-580-4_11 20652511

[ece370064-bib-0026] Lemanik, A. , Nadachowski, A. , & Socha, P. (2022). Biostratigraphic significance of the root vole (*Alexandromys oeconomus*) for dating late middle and early late Pleistocene (MIS 8‐MIS 3) Neanderthal Sites in Southern Poland. Archaeological and Anthropological Sciences, 14(6), 1–14. 10.1007/s12520-022-01580-9

[ece370064-bib-0027] López‐García, J. M. , Cuenca‐Bescós, G. , Galindo‐Pellicena, M. Á. , Luzi, E. , Berto, C. , Lebreton, L. , & Desclaux, E. (2021). Rodents as indicators of the climatic conditions during the middle Pleistocene in the southwestern Mediterranean region: Implications for the environment in which hominins lived. Journal of Human Evolution, 150, 102911. 10.1016/j.jhevol.2020.102911 33254079

[ece370064-bib-0028] Luzi, E. (2018). *Morphological and morphometric variations in middle and late Pleistocene Microtus arvalis and Microtus agrestis populations: Chronological insight, evolutionary trends and palaeoclimatic and palaeoenviromental inferences* (Doctoral Thesis, Universitat Rovira i Virgili), 138 p. http://hdl.handle.net/10803/586081

[ece370064-bib-0029] Luzi, E. , & López‐García, J. M. (2017). Patterns of variation in Microtus arvalis and Microtus agrestis populations from middle to late Pleistocene in southwestern Europe. Historical Biology, 31(5), 535–543. 10.1080/08912963.2017.1375490

[ece370064-bib-0031] Miele, V. , Dussert, G. , Cucchi, T. , & Renaud, S. (2020). Deep learning for species identification of modern and fossil rodent molars.

[ece370064-bib-0032] Moclán, A. , Domínguez‐García, Á. C. , Stoetzel, E. , Cucchi, T. , Sevilla, P. , & Laplana, C. (2023). Machine learning interspecific identification of mouse first lower molars (genus Mus Linnaeus, 1758) and application to fossil remains from the Estrecho cave (Spain). Quaternary Science Reviews, 299, 107877. 10.1016/j.quascirev.2022.107877

[ece370064-bib-0033] Nadachowski, A. (1991). Systematics, geographic variation, and evolution of snow voles (chionomys) based on dental characters. Acta Theriologica, 36, 1–45. 10.4098/at.arch.91-1

[ece370064-bib-0034] Navarro, N. , Montuire, S. , Laffont, R. , Steimetz, E. , Onofrei, C. , & Royer, A. (2018). Identifying past remains of morphologically similar vole species using molar shapes. Quaternary, 1(3), 20. 10.3390/quat1030020

[ece370064-bib-0035] Nefedov, A. (2016). Support vector machines: A simple tutorial . [Online], https://sustech‐cs‐courses.github.io/IDA/materials/Classification/SVM_tutorial.pdf, tanggal akses

[ece370064-bib-0036] Obi, J. C. (2017). A comparative study of the Fisher's discriminant analysis and support vector machines. European Journal of Engineering and Technology Research, 2(8), 35–40.

[ece370064-bib-0037] Pardiñas, U. F. J. , Myers, P. , Léon‐Paniagua, L. , Ordóñez, G. N. , Cook, J. A. , Kryštufek, B. , Haslauer, R. , Bradley, R. D. , Shenbrot, G. I. , & Patton, J. L. (2017). Family Cricetidae (true hamsters, voles, lemmings and New World rats and mice). In D. E. Wilson , T. E. Lacher , & R. A. Mittermeier (Eds.), Handbook of the mammals of the world, Rodents II (Vol. 7, pp. 204–535). Lynx Edicionas.

[ece370064-bib-0038] Peña, D. (2002). Análisis de datos multivariantes (p. 391). Mc GRAW‐HILL /INTERAMERICANA DE España, S.A.U. Página.

[ece370064-bib-0039] Piras, P. , Marcolini, F. , Claude, J. , Ventura, J. , Kotsakis, T. , & Cubo, J. (2012). Ecological and functional correlates of molar shape variation in European populations of Arvicola (Arvicolinae, Rodentia). Zoologischer Anzeiger ‐ A Journal of Comparative Zoology, 251(4), 335–343. 10.1016/j.jcz.2011.12.002

[ece370064-bib-0040] Quenu, M. , Trewick, S. A. , Brescia, F. , & Morgan‐Richards, M. (2020). Geometric morphometrics and machine learning challenge currently accepted species limits of the land snail Placostylus (Pulmonata: Bothriembryontidae) on the isle of pines, New Caledonia. Journal of Molluscan Studies, 86(1), 35–41. 10.1093/mollus/eyz031

[ece370064-bib-0041] Román, J. (2019). *Manual para la identificación de los cráneos de los roedores de la península ibérica, islas baleares y canarias*. Sociedad Española Para la Conservación y Estudio de los Mamíferos.

[ece370064-bib-0042] Sasikanth . (2022). Modified Hausdorff distance MATLAB central file exchange . Recuperado September 9, 2022. https://www.mathworks.com/matlabcentral/fileexchange/29968‐modified‐hausdorff‐distance

[ece370064-bib-0043] Smola, A. J. (1996). Regression estimation with support vector learning machine . (MSc Thesis) Technische Universität München.

[ece370064-bib-0044] The MathWorks Inc . (2017). MATLAB version: 9.3.0.713579 (R17b). The MathWorks Inc. https://www.mathworks.com

[ece370064-bib-0045] Van der Meulen, A. J. (1973). Middle Pleistocene smaller mammals from the Monte Peglia, (Orvieto, Italy) with special reference to the phylogeny of Microtus (Arvicolinae, Rodentia) (p. 144). QUATERNARIA.

